# Supplementary material for: Targeting translation initiation yields fast-killing therapeutics against the zoonotic parasite Cryptosporidium parvum
Source: PLoS Pathog. 2025 Jul 28;21(7):e1012881. doi: 10.1371/journal.ppat.1012881 (PMC12313074; doi:10.1371/journal.ppat.1012881)
Supplement: S1 Table — (PDF) [file ppat.1012881.s001.pdf]

S1 Table. Transcript abundance comparison of *CpeIF4A* gene with *CpeIF4A1* and one of the DEAD-box (DDX) domain-containing proteins clustered with DDX19/25 proteins (cgd8_4750)*

Sample	CpeIF4A (cgd1_880)		CpeIF4A1 (cgd7_3940)		DDX19/25 (cgd8_4750)	
	TPM	Fold	TPM	Fold	TPM	Fold
Oocysts- sense - unique	4,265.2	1.0	58.0	-73.5	165.5	-25.8
Pooled in vitro infection samples - unique	6,239.5	1.0	125.3	-49.8	355.9	-17.5
Asexual - unique	2,422.4	1.0	42.1	-57.6	114.1	-21.2
Female in vitro culture - unique	600.9	1.0	7.3	-82.1	22.8	-26.4
Female in vivo mouse - unique	1,046.5	1.0	14.3	-73.3	31.6	-33.1
Sporozoites - unique	8,893.7	1.0	150.5	-59.1	233.8	-38.0
24 hr culture - unique	1,028.5	1.0	32.2	-31.9	112.2	-9.2
48 hr culture - unique	466.3	1.0	14.6	-31.9	48.3	-9.7

*Transcriptomic data were extracted from CryptoDB (https://cryptodb.org/). Transcript abundance is expressed as transcripts per million (TPM). Fold changes were calculated using CpeIF4A (cgd1_880) as the baseline. Positive values indicate fold increases relative to CpeIF4A, while negative values indicate fold decreases.
