# Supplementary material for: Targeting translation initiation yields fast-killing therapeutics against the zoonotic parasite Cryptosporidium parvum
Source: PLoS Pathog. 2025 Jul 28;21(7):e1012881. doi: 10.1371/journal.ppat.1012881 (PMC12313074; doi:10.1371/journal.ppat.1012881)
Supplement: S2 Table — (PDF) [file ppat.1012881.s002.pdf]

S2 Table. Proteomic abundance comparison of CpeIF4A with CpeIF4A1 and one of the DEAD-box (DDX) domain-containing proteins clustered with DDX19/25 proteins (cgd8_4750)*

Protein name	Total sequences		Unique sequences		Sum spectra	
	Count	Fold	Count	Fold	Count	Fold
CpeIF4A (cgd1_880)	53	1.0	26	1.0	115	1.0
CpeIF4A1 (cgd7_3940)	1	-53.0	1	-26.0	1	-115.0
DDX19/25 (cgd8_4750)	9	-5.9	5	-5.2	10	-11.5

*Proteomic data were sourced from CryptoDB (https://cryptodb.org/). Proteomic abundances are combined mass spectrum counts from various oocyst and sporozoite samples as described in the CryptoDB. Fold changes were calculated using CpeIF4A (cgd1_880) as the baseline. Positive values indicate fold increases relative to CpeIF4A, while negative values indicate fold decreases.
