# Supplementary material for: Targeting translation initiation yields fast-killing therapeutics against the zoonotic parasite Cryptosporidium parvum
Source: PLoS Pathog. 2025 Jul 28;21(7):e1012881. doi: 10.1371/journal.ppat.1012881 (PMC12313074; doi:10.1371/journal.ppat.1012881)
Supplement: S3 Table — (PDF) [file ppat.1012881.s003.pdf]

S3 Table. Mouse health scoring scales

Parameters	Score		
	0	1	2
**Fur condition**	**Normal**<br>Smooth and shiny, no frizz.	**Moderate**<br>Slightly frizzy, generally less shiny.	**Severe**<br>Messy and frizzy; has lost luster.
**Body weight**	**Normal to 1% weight loss**<br>Daily weight is increased, unchanged, or decreased by <1%.	**Slight to moderate**<br>Daily weight loss between 1% and 10%.	**Severe weight loss**<br>Daily weight loss >10%.
**Hunchbackedness**	**Normal**<br>Active movement and elongated posture.	**Slight to moderate**<br>Not particularly active, their backs slightly arched. They may sway from side to side when they walk.	**Abnormal**<br>Severe arching of the back and reluctance to move.
**Behavior**	**Normal**<br>Paying attention to caretakers; actively reacting when caretaker's hands enters the cage.	**Mildly to moderately depressed**<br>Paying some attention to caretakers; Slow moving in response to visitor's touch to cages.	**Severely depressed**<br>Paying no attention to caretakers; No or little moving even when being touched.

References:

Whitehead JC, Hildebrand BA, Sun M, Rockwood MR, Rose RA, Rockwood K, et al. A clinical frailty index in aging mice: comparisons with frailty index data in humans. J Gerontol A Biol Sci Med Sci. 2014;69(6):621-32. Epub 2013/09/21. doi: 10.1093/gerona/glt136. PubMed PMID: 24051346; PubMed Central PMCID: PMCPMC4022099.

Shrum B, Anantha RV, Xu SX, Donnelly M, Haeryfar SM, McCormick JK, et al. A robust scoring system to evaluate sepsis severity in an animal model. BMC Res Notes. 2014;7:233. Epub 2014/04/15. doi: 10.1186/1756-0500-7-233. PubMed PMID: 24725742; PubMed Central PMCID: PMCPMC4022086.
