# Supplementary material for: Targeting translation initiation yields fast-killing therapeutics against the zoonotic parasite Cryptosporidium parvum
Source: PLoS Pathog. 2025 Jul 28;21(7):e1012881. doi: 10.1371/journal.ppat.1012881 (PMC12313074; doi:10.1371/journal.ppat.1012881)

Supporting information (S1 Fig)

S1 Fig. Transient over-expression of MDR1 in host cells as detected by immunofluorescence assay (IFA) using an anti-MDR1 antibody. In this in vitro model, HCT-8 cells were transiently transfected with with pCVM3 vector carrying *MDR1* gene (pCMV3-MDR1; marked as HCT-8/MDR1). Negative control cells were transfected with blank pCMV3 vector (marked as HCT-8/NC). Both cell types were labeled with ant-MDR1 antibody at the same concentration, and images were taken with the same exposure time.

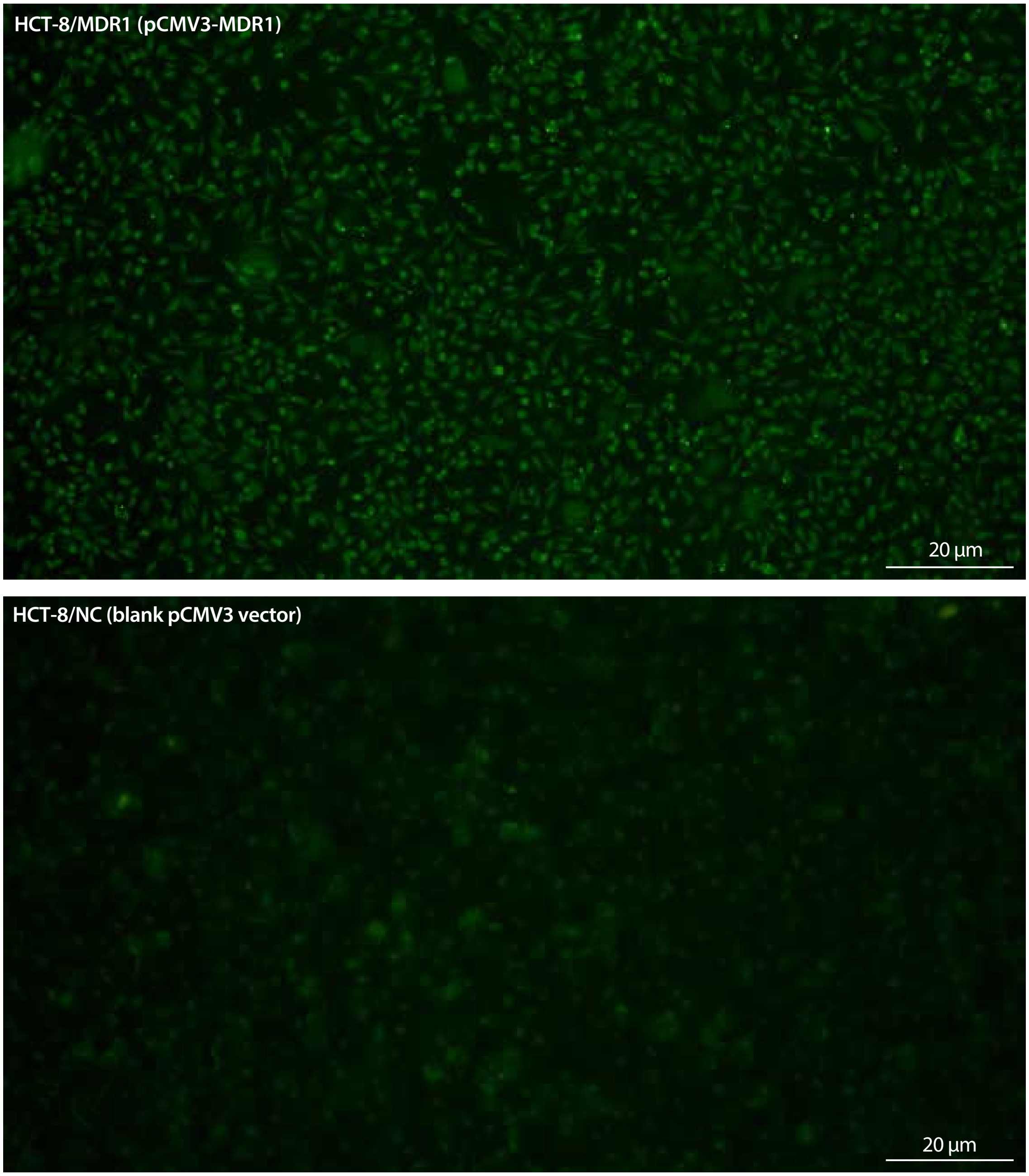

Supplement: S1 Fig — In this in vitro model, HCT-8 cells were transiently transfected with pCVM3 vector carrying MDR1 gene (pCMV3-MDR1; marked as HCT-8/MDR1). Negative control cells were transfected with blank pCMV3 vector (marked as HCT-8/NC). Both cell types were labeled with ant-MDR1 antibody at the same concentration, and images were taken with the same exposure time. (PDF) [file ppat.1012881.s005.pdf]
