# Supplementary material for: Targeting translation initiation yields fast-killing therapeutics against the zoonotic parasite Cryptosporidium parvum
Source: PLoS Pathog. 2025 Jul 28;21(7):e1012881. doi: 10.1371/journal.ppat.1012881 (PMC12313074; doi:10.1371/journal.ppat.1012881)

Supporting information (S2 Fig)

S2 Fig. Evaluation of the drug tolerance in mice to Roc-A. C57BL/6 mice (8-wk old; 3 mice/group) were administered by oral gavage with Roc-A at 0 (1% DMSO vehicle), 0.5, 1.0 and 2.0 mg/kg/d in a single daily dose for 7 days. **(A)** Percent daily weight gains in mice from 0 to 7 dpa (day post-administration). Bars indicate standard error of the mean (SEM). * = p <0.05, ** = p <0.01, *** = p <0.001, and **** = p <0.0001 by Tukey's multiple comparison test (vs. vehicle control). **(B)** Mouse health scores calculated based on the survival, weight gains, fur condition, hunchbackedness, and animal attitude scales (see Materials and Methods for detail).

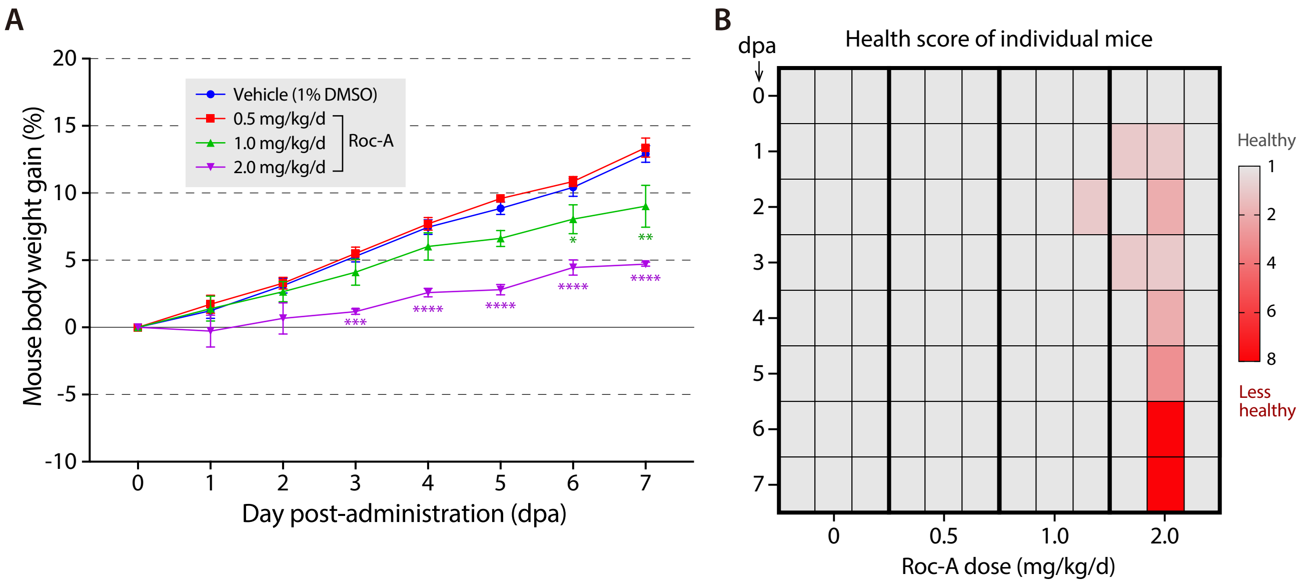

Supplement: S2 Fig — C57BL/6 mice (8-wk old; 3 mice/group) were administered by oral gavage with Roc-A at 0 (1% DMSO vehicle), 0.5, 1.0 and 2.0 mg/kg/d in a single daily dose for 7 days. (A) Percent daily weight gains in mice from 0 to 7 dpa (day post-administration). Bars indicate standard error of the mean (SEM). * = p < 0.05, ** = p < 0.01, *** = p < 0.001, and **** = p < 0.0001 by Tukey’s multiple comparison test (vs. vehicle control). (B) Mouse health scores calculated based on the survival, weight gains, fur condition, hunchbackedness, and animal attitude scales (also see Materials and Methods for detail). (PDF) [file ppat.1012881.s006.pdf]
