# Supplementary material for: Targeting translation initiation yields fast-killing therapeutics against the zoonotic parasite Cryptosporidium parvum
Source: PLoS Pathog. 2025 Jul 28;21(7):e1012881. doi: 10.1371/journal.ppat.1012881 (PMC12313074; doi:10.1371/journal.ppat.1012881)

Supporting information (S3 Fig)

S3 Fig. Phylogenetic tree of CpeIF4A and closely related orthologs from various *Cryptosporidium* species, selected apicomplexan species, and humans. The displayed is a consensus tree inferred by Bayesian inference (BI) analysis, which separates the sequences into seven clusters. Six clusters contain human orthologs, including eIF4A-I/II, eIF4A-III, DDX19/25, DDX39, DDX6, and DDX5/17. The seventh cluster appears unique to *Cryptosporidium* (absent in humans and other apicomplexans), but closely related to DDX5/17 subfamily proteins.

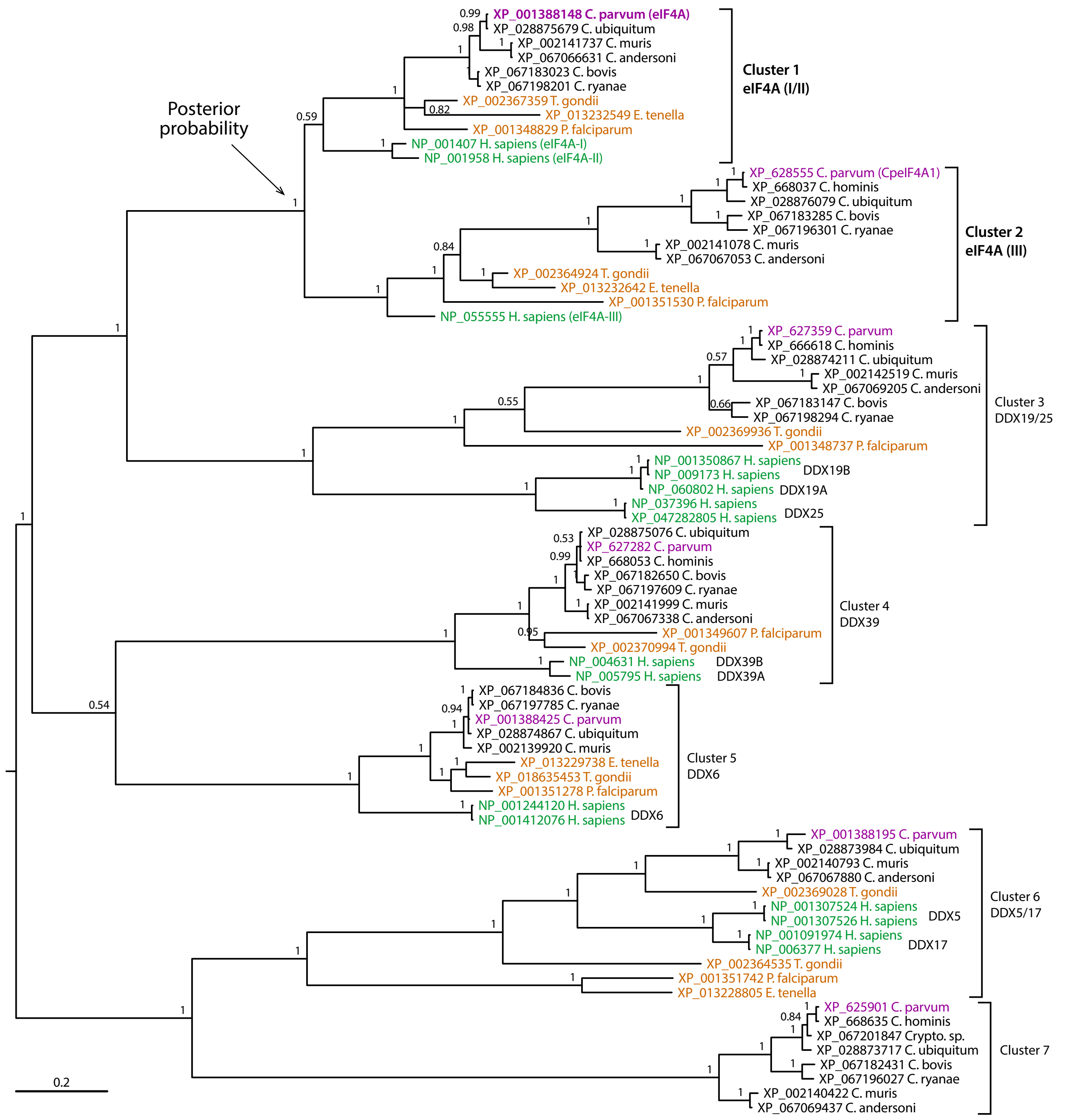

Supplement: S3 Fig — The displayed is a consensus tree inferred by Bayesian inference (BI) analysis, which separates the sequences into seven clusters. Six clusters contain human orthologs, including eIF4A-I/II, eIF4A-III, DDX19/25, DDX39, DDX6, and DDX5/17. The seventh cluster appears unique to Cryptosporidium (absent in humans and other apicomplexans), but closely related to DDX5/17 subfamily proteins. (PDF) [file ppat.1012881.s007.pdf]
