# Supplementary material for: Targeting translation initiation yields fast-killing therapeutics against the zoonotic parasite Cryptosporidium parvum
Source: PLoS Pathog. 2025 Jul 28;21(7):e1012881. doi: 10.1371/journal.ppat.1012881 (PMC12313074; doi:10.1371/journal.ppat.1012881)

Supporting information (S4 Fig)

S4 Fig. Immunostaining of CpeIF4A in intracellular *C. parvum* cultured with HCT-8 cells. Host cell monolayers infected with *C. parvum* for 11 h were fixed with paraformaldehyde (4%) and stained with affinity-purified anti-CpeIF4A antibody. Nuclei (Nuc) were counterstained with DAPI. The images show that CpeIF4A is distributed in the cytoplasm with stronger signals under the plasma membrane in varied developmental stages during merogony.

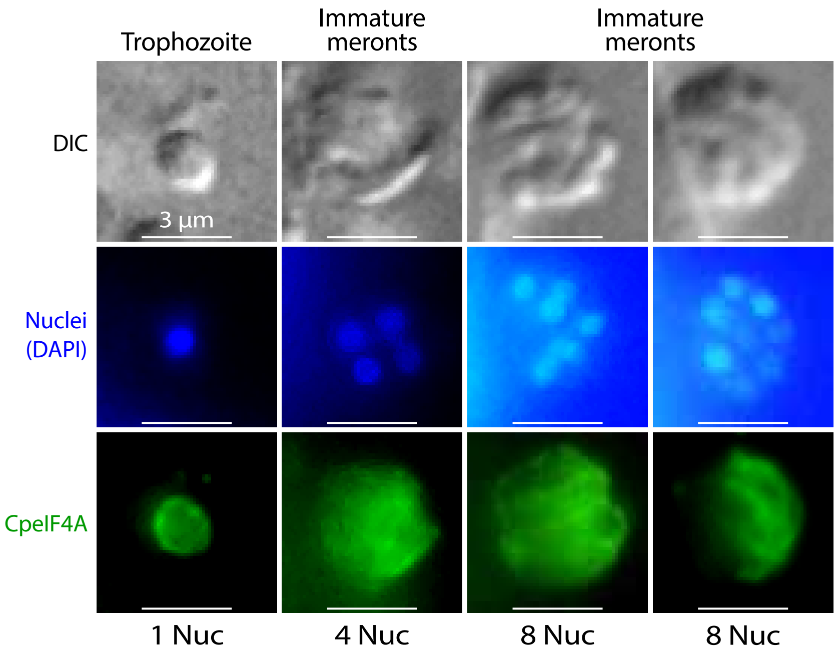

Supplement: S4 Fig — Host cell monolayers infected with C. parvum were fixed with paraformaldehyde (4%) and stained with affinity-purified anti-CpeIF4A antibody. Nuclei (Nuc) were counterstained with DAPI. The images show that CpeIF4A is distributed in the cytoplasm with stronger signals under the plasma membrane in varied developmental stages during merogony. (PDF) [file ppat.1012881.s008.pdf]
