# Supplementary material for: Targeting translation initiation yields fast-killing therapeutics against the zoonotic parasite Cryptosporidium parvum
Source: PLoS Pathog. 2025 Jul 28;21(7):e1012881. doi: 10.1371/journal.ppat.1012881 (PMC12313074; doi:10.1371/journal.ppat.1012881)

Supporting information (S5 Fig)

S5 Fig. Structural model of CpeIF4A superimposed with human eIF4A-I in complex with the ATP analog AMPPNP, Roc-A and polypurine RNA. CpeIF4A structure is predicted by Swiss-Model and superimposed with the crystal structure of the human eIF4A1 in complex with AMPPNP, polypurine RNA and Roc-A (PDB entry: 5ZC9). The superimposed structure shows high structural similarity, but subtle difference, between CpeIF4A and human eIF4A-I, predicting the binding of Roc-A at the "bi-molecular cavity" formed by eIF4A-I and a sharply bent pair of purines in the RNA. Also see **Fig. 6** for more detailed comparison at the Roc-A binding surface.

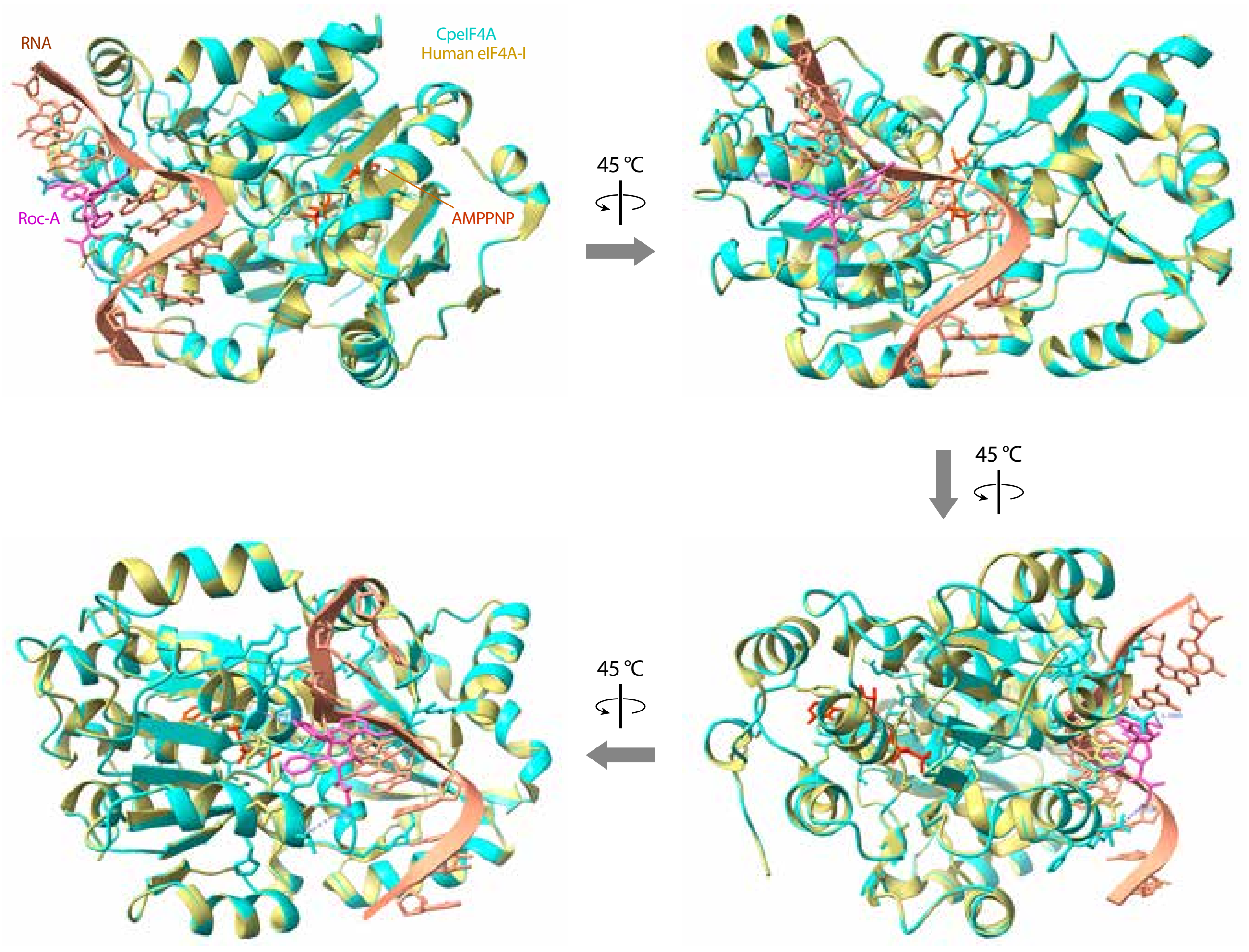

Supplement: S5 Fig — CpeIF4A structure is predicted by Swiss-Model and superimposed with the crystal structure of the human eIF4A1 in complex with AMPPNP, polypurine RNA and Roc-A (PDB entry: 5ZC9). The superimposed structure shows high structural similarity, but subtle difference, between CpeIF4A and human eIF4A-I, predicting the binding of Roc-A at the “bi-molecular cavity” formed by eIF4A-I and a sharply bent pair of purines in the RNA. Also see Fig 6 for more detailed comparison at the Roc-A binding surface. (PDF) [file ppat.1012881.s009.pdf]
