# Supplementary material for: Targeting translation initiation yields fast-killing therapeutics against the zoonotic parasite Cryptosporidium parvum
Source: PLoS Pathog. 2025 Jul 28;21(7):e1012881. doi: 10.1371/journal.ppat.1012881 (PMC12313074; doi:10.1371/journal.ppat.1012881)
Supplement: Attachment — Submitted filename: Point2point_responses01b.pdf [file ppat.1012881.s011.pdf]

Point-to-point responses to the reviewers' comments

PPATHOGENS-D-25-00012

Introduction: We express our gratitude to the editor and reviewers for their enthusiasm and constructive critiques, which have significantly strengthened the manuscript.

In response, we have conducted two additional experiments: (i) an extended 30-day study in NCG mice to monitor oocyst shedding for 15 days post-treatment, addressing concerns about recrudescence; and (ii) structure-guided mutagenesis of CpeIF4A (F161L, I197M, and the double mutant), followed by enzymology and Roc-A binding assays. Furthermore, we have expanded the Discussion section to include recent research on rocaglates, clarified structural differences between parasite and human eIF4A, and corrected minor textual issues throughout.

The manuscript has undergone substantial revisions to enhance clarity and address textual corrections. Below are our point-by-point responses to the comments raised by the editor and reviewers:

Comment #1

Editor comments:

The reviewers identified several key weaknesses that need to be addressed to strengthen the manuscript. First, there is insufficient discussion of RocA's effects on mammalian cells, its broad anti-pathogen activity, and the potential for structural differences between eIF4A proteins in Cryptosporidium and humans that could guide specificity improvements.

Additionally, the novelty and selectivity of RocA for CpeIF4A are overstated, requiring a deeper exploration of its broader activity and other potential targets in Cryptosporidium.

Response:

We have added more discussion on the effects of Roc-A on mammalian cells and broad anti-pathogen activity. We have also re-built the structural model of CpeIF4A in comparison with human eIF4A, and identified several amino acid replacements between parasite and human eIF4A proteins, which could be explored to guide specificity improvement of Roc-A. Other potential targets in the parasite are also discussed.

Comment #2

Lastly, the in vivo mouse data lacks evaluation of recrudescence post-treatment, and the manuscript would benefit from leveraging established Cryptosporidium mouse models and/or resistance-conferring mutation studies to validate CpeIF4A as the target and assess the compound's long-term efficacy.

Response:

We have performed new 30-day mouse experiment and evaluated post-treatment recrudescence and long-term efficacy. Please see more details in our responses below.

Comment #3

Essentiality of CpeIF4A for parasite does not need to experimentally demonstrated however literature on this factor in other apicomplexan parasites should be more comprehensively covered.

Response:

Thanks for your comment and understanding. We have expanded our discussion on the importance of eIF4A and referenced published studies on apicomplexan eIF4A proteins.

At present, we are optimizing protocols for conditional gene knockout in *C. parvum*. The genetic manipulation of CpeIF4A and related DDX proteins will be incorporated into our next phase of research to study their biological roles in the parasite. We look forward to sharing our new findings in the near future.

Part I - Summary
Please use this section to discuss strengths/weaknesses of study, novelty/significance, general execution and scholarship.

Comment #4:

Reviewer #1: Meng Li et al. found that C. parvum eukaryotic initiation factor 4A (CpeIF4A) could be a target for the development of fast-killing anti-cryptosporidial therapeutics. In a series of experiments, they showed that Roc-A is a specific inhibitor of eIF4A and is highly effective against C. parvum growth in vitro and in vivo. They also found that Roc-A tightly binds to the CpeIF4A-RNA complex and suppresses protein synthesis in the parasite.

Overall, the manuscript provides a novel drug target, CpeIF4A, and a promising anti-cryptosporidial drug, Roc-A. It paves the way for the discovery of new anti-cryptosporidial drugs. There are still some suggestions for specific points shown below that may be useful to improve the manuscript.

Response:

We appreciate the overall positive comments! Other comments from the reviewer have been addressed in the part II and III below.

Comment #5:

Reviewer #2: The manuscript by Li et al describes the inhibition of Cryptosporidium parvum eukaryotic initiation factor 4A (CpeIF4A) by Rocaglamide-A (RocA) compound using biochemical, in vitro and in vivo assays. The topic of this study is important and timely since it is imperative to

discover new drug targets for the development of much needed effective drugs against cryptosporidiosis. The authors provide strong biochemical data demonstrating the inhibition of CpeIF4A activity by RocA, disruption of protein synthesis in sporozoites, as well as the quick parasite killing potential of this compound in vitro at nanomolar concentration. Additionally, they tested this compound in IFN-g KO mice and found a reduction in oocyst production.

Response:

We appreciate the overall positive comments!

Comment #6:

Although these results are interesting, they are preliminary and several additional experiments are required to fully establish that Roc-A is a selective and specific inhibitor that is only targeting Cryptosporidium eIF4A. The in vivo data is not rigorous as no intestinal histological analysis has been provided, and the infection was monitored for only a limited time after the end of the drug-treatment period.

Response:

Thanks for the comments and suggestions! Similar concern on the limited post-treatment time in the in vivo efficacy assay is also raised by reviewer 3 (comment #8).

The comment have been well taken. As suggested, we have performed new experiments to evaluate potential post-treatment relapse in NCG mice, and mutagenesis of CpeIF4A to evaluate the effects of two key amino acids on the inhibition and binding of Roc-A. These are described in more detail in part II below.

Comment #7

Furthermore, the authors do not cite or discuss studies that have previously reported anti-parasitic activities of RocA in other parasites such as Leishmania and Plasmodium (PMID:36144626; PMID: 21417924). RocA has been reported to be an inhibitor of prohibitin and has been reported to target both asexual-blood stage and gametocyte stages in the malaria parasite (PMID: 37088154). The lack of mention/discussion about these relevant studies on anti-parasitic potential of RocA, and the possibility that this compound may be hitting other targets prohibitin or other RNA helicases) limits the novelty of this work. Additional experiments are therefore required to validate this drug target in Cryptosporidium.

Response:

This is indeed our ignorance. We have cited and included more discussion on previously studies related to the anti-parasitic activities of Roc-A.

Comment #8:

Reviewer #3: Very interesting and exciting study reporting that a natural product, rocaglamide A (Roc-A) targeting the translation initiation factor eIF4A has potential as an anti-cryptosporidium

therapeutic. The compound has low nM efficacy, very high selectivity indices, appears to specifically target the Cp eIF4A and is very effective against Cryptosporidium in IFN-gamma knock out mice. eIF4A is an RNA helicase, and the authors demonstrate that Roc-A inhibits the helicase activity of eIF4a, and binds with high affinity to eIF4A in the presence of RNA and ATP. Moreover they show that RocA inhibits protein synthesis in encysted sporozoites. Overall the study is well described, and the experiments are rigorous.

My major concerns are that the mouse experiment ends only 24 hours after the last treatment so there is no evaluation of the possibility of recrudescence, and overall there needs to be more in depth discussion of Roc-A's activities against human eIF4A and comparison to the results presented here, as well as a discussion of the feasibility of developing a lead compound with better selectivity for Cp over mammalian cells (based on the toxicity in mice)

Response:

We appreciate the overall positive comments! The comment on the limited post-treatment time in the in vivo efficacy assay is also raised by reviewer 2 (comment #6).

As suggested, we have performed new experiments to evaluate potential post-treatment relapse in NCG mice, and mutagenesis of CpeIF4A to evaluate the effects of two key amino acids on the inhibition and binding of Roc-A. These are described in more detail in part II below.

Part II – Major Issues: Key Experiments Required for Acceptance

Comment #9:

Reviewer #1: 1. In Figures 4B and C, you have shown the alignment of CpeIF4A1 and CpeIF4A2 with other orthologs of different species. In the manuscript and figure legends, you only mentioned CpeIF4A and CpeIF4A1. I am struggling to find the information about the CpeIF4A2. Please make sure that these two figures are correctly labelled or described.

Response:

We apologize for the error in Fig. 4B and 4C. The labels should be CpeIF4A (formerly CpeIF4A1) and CpeIF4A1 (formerly CpeIF4A2). This has been corrected in the revised Fig. 4.

Note: We named cgd1_880 as CpeIF4A and cgd7_3940 as CpeIF4A1 to align with CryptoDB and GenBank annotations.

Comment #10:

Reviewer #2: Further experiments are required to establish that Roc-A is a selective and specific inhibitor of CpeIF4A. Although this gene may be likely essential for parasite survival, it needs to be experimentally validated using genetic approaches.

Response:

The indispensability of CpeIF4A could be assessed through genetic manipulation, which is now feasible for *C. parvum*, although it remains less straightforward compared to *Toxoplasma* and some other organisms. Our laboratory has recently created transgenic *C. parvum* strains with tagged genes. We are also investigating the knockout (KO) or conditional KO of target genes, including CpeIF4A and CpeIF4A1, as part of our ongoing research. Given that performing conditional gene knockout for essential genes requires considerable time, we will present our findings related to CpeIF4A and associated genes in our subsequent studies.

Comment #11:

In the mammalian literature, it has been shows that Phe163Leu-Ile199Met mutations in eIF4A1 confer RocA-resistance in HEK293 cells (PMC3796129, PMC6386617). Are any of these mutations conserved in CpeIF4A that can be mutated for RocA resistance studies and genetic validation of this target? Is it possible that Roc-A has other targets in Cryptosporidium such as other DEAD-box RNA helices, prohibitin etc.?

Response:

Thank you for the suggestion and comment. Reviewer #3 has made similar remarks regarding the residues conferring resistance (comment #16).

1) Mutations vs. Roc-A resistance: Three CpeIF4A mutations have been generated and their RNA helicase activity, sensitivity to Roc-A, and binding affinity to Roc-A were evaluated. Single or double mutations (Ile197Met and/or Phe161Leu) only slightly altered the helicase activity (Fig. 4J, inset), but significantly increased resistance to Roc-A (2.3, 21.4, and 38.7-fold increases of IC50 values, see Fig. 4J and 4K). The mutations also substantially reduced binding affinity to Roc-A (Fig. 5E and 5F).

2) Other potential targets for Roc-A: Roc-A may affect other DDX helicases. Based on sequence similarity at the Roc-A binding site, CpeIF4A1 is likely another target. This was briefly mentioned in the original submission, and has been further elaborated in the revised manuscript.

Comment #12:

There are acute and chronic (NOD-SCID-gamma) mouse models of cryptosporidiosis and reporter parasites available to test the efficacy of compounds and monitor any relapse after the compound administration period is over. These model systems and in vivo efficacy assays are now typically used in the Cryptosporidium research field. Therefore, testing the Roc-A inhibitor using any one of these models would provide strong evidence on the in vivo efficacy of this compound and guide future compound optimization and efficacy studies.

Response:

Reviewers #2 and #3 provided similar comments and suggestions. In response, a new in vivo assay was conducted to observe potential post-treatment relapse. Immunodeficient NCG mice, comparable to NSG mice, were used due to their timely availability. The new experiment lasted for

a total of 30 days, including 10 days to establish chronic infection, 5 days of Roc-A treatment (vs. vehicle), and 15 days to observe post-treatment relapse.

The results were consistent with those obtained in the first in vivo assay during the first 15 days, showing a significant decrease in oocyst shedding upon Roc-A treatment and no rebound during the subsequent 15-day post-treatment period. New data from this experiment have been included in the manuscript, along with new figures (Fig. 3D to 3F) and a table (Table 2).

The suggestion to include intestinal histopathology was noted. However, the institutional histology core is currently undergoing renovation, resulting in a backlog that could delay resubmission beyond the first author's graduation deadline. Given that two independent mouse models demonstrate rapid (>70–90%) and sustained suppression of oocyst shedding with no rebound during a 15-day follow-up, it is concluded that additional histology is not absolutely essential to support the study's conclusions. Histological analysis will be incorporated in future work once the facility reopens.

Comment #13:

Reviewer #3: 1. Fig 3: The mouse experiment demonstrates the impressive rapid action of Roc A against Cp infection, but there is no evaluation of the possibility of recrudescence once treatment with RocA is stopped (final samples are taken 24 hours after last administration of drug). This is a concern as the RocA dose is extraordinarily low (0.5 mg/kg).

Response:

Both reviewers #2 and #3 provided similar comment and suggestion. As advised, we have performed new in vivo assay to observe potential post-treatment relapse. Please see our reply to the comment #12 above.

Comment #14:

2. It would be helpful in the introduction/discussion to have more information on the effects of RocA on mammalian cells. A quick glance at PubMed reveals that there is a significant body of information on Roc-As effects on mammalian cells. While the in vitro selectivity indices are excellent, the toxicity in the mice is concerning, and the literature could provide insight into why this compound is so toxic in mice.

Response:

As suggested, we have added more discussions on the effect of Roc-A on mammalian cells and in vivo toxicity.

Comment #15:

Supplemental Fig 4A shows CpeIF4A predicted structure overlaid on the crystal structure of human eIF4A1-ATP-ROC-RNA complex, but there is little discussion of the potential to improve the specificity of the compound for Cp, beyond mentioning that the two IF4s have high structural similarity.

Response:

Good suggestion! Our collaborator and coauthor of the manuscript Dr. Pingwei Li, who is a structural biologist, has rebuilt the CpeIF4A model to compare Roc-A binding surfaces between CpeIF4A and human eIF4A (HseIF4A). We identified three amino acid replacements on the Roc-A binding surface: N167, R168, and D194 in HseIF4A are replaced by D165, K166, and V192 in CpeIF4A. Modifying Roc-A to enhance interactions with nonpolar V192 and acidic D165 may improve binding specificity. Details are discussed in the Results section "Structural modeling and mutagenesis support the high-affinity binding of Roc-A to the CpeIF4A-RNA complex and the potential to improve the binding specificity". Refer to **Fig. 6** for surface comparisons.

Comment #16:

The manuscript would be greatly enriched by a more in depth discussion of this compound's activities against the human eIF4 and how this compares to the activity against CpeIF4A. For example, residues that confer resistance to RocA are found near the RNA binding motif (Sadlish, 2013). Are there resistance conferring residues in any of the apicomplexan sequences?

Response:

We have performed new experiments, in which three CpeIF4A mutations (i.e., F161L, I197M and double mutation F161L+I197M) were produced and tested the effects on helicase activity, sensitivity to Roc-A, and binding affinity. Also see response to comment #11 above.

Part III – Minor Issues: Editorial and Data Presentation Modifications

Comment #17:

Reviewer #1: 1. In figures 2C and 2D, why do you use 3 nM Roc-A to the culture, is it the value of EC90? Could you clarify the reason for using 3 nM Roc-A?

Response:

It is the concentration at ~EC63 (absolute EC value based on the plot of 44-h infection assay). At this concentration, smaller reductions in the "effect on developmental stage assay" could be better quantified. This has been clarified in the revised manuscript.

Comment #18:

2. In Figure 2D, you treated Roc-A in culture for 3-10 h or 3-22 h, and both treatments could inhibit C. parvum growth on HCT-8 cells. But for these two treatment time points, the drug was effective on the asexual life stage of C. parvum. I was wondering if you have tried adding Roc-A at the sexual life stage (e.g., 30-44 h) to see if the Roc-A could still block parasite growth or not?

Response:

We have included treatment of Roc-A at the sexual stage of *C. parvum* (30-44 h), in which the activity is relatively low (i.e., ~20% reduction of parasite load) (**Fig. 2D**).

We speculate that the lower activity may be attributable to the lower activity of protein synthesis (as both macrogametes and microgametes are relatively inactive and unable to fertilize to form zygotes) and/or the potentially lower permeability of Roc-A. This has been discussed in the revised manuscript.

Comment #19:

3. For the in vivo drug efficacy test (Figure 3C), is the data from one or two individual mouse experiments?

Response:

The 15-day in vivo experiments and the newly performed 30-day experiment were each performed once (total two in vivo experiments). This is clarified in the Materials and Methods section under the subtitle "Data analysis and statistics".

Comment #20:

4. In Figure 4F, it would be better to add scale bars to the images.

Response:

Scale bars are added.

Comment #21:

5. Why did you choose sporozoites for the RNA-seq assay? Based on Figure 6B, I don't think there are significant differences in fluorescence signal when you treat sporozoites with/without Roc-A for 2 h. In addition, the transcription level in sporozoites is relatively lower than in other parasite stages. CpeIF4A is present at relatively high levels in all parasite life stages (Figure 4D). Have you tried RNA-seq in intracellular parasites? Maybe you should try to infect Cp on HCT-8 cells, treat with/without Roc-A for a few hours, and then do the RNA-seq. It might give you more candidates to analysis.

Response:

Sporozoites are the only pure parasite material obtainable. While it is possible to isolate total mRNA from intracellular parasites, the proportion of parasite mRNA is exceedingly low compared to host cell mRNA (less than 0.02% based on our unpublished calculation). Consequently, without suitable enrichment, achieving the necessary sequencing depth for quantitative analysis of intracellular parasite transcriptomes would be challenging. The current enrichment protocols are invasive and could potentially alter parasite transcription, making them unsuitable for accurate quantitative analysis.

Note: We are actually working on an enrichment protocol that does not affect transcriptional profiles. We hope that the protocol will be optimized in the near future to facilitate cost-effective, reliable quantitative analysis of intracellular parasite transcriptomes.

Comment #22:

6. Has eukaryotic initiation factor 4A been studied in other apicomplexan parasites? It would be better to add some background about the eukaryotic initiation factor 4A from other apicomplexan parasites in the introduction or discussion section.

Response:

There are about 15 articles on apicomplexan eIF4A in PubMed, some with functional analysis discussed in the Discussion section.

Comment #23:

Reviewer #2: Line 127: HapG2 to be changed to 'HepG2'
Line 523: MUSCL change to 'MUSCLE'

Response:

All typographical errors have been addressed. Additionally, the manuscript has undergone comprehensive revisions to enhance clarity and rectify textual issues.

Comment #24:

Reviewer #3: Line 127: The approximate selectivity index should be 56,000 not 560,000.

Response:

Corrections are made in the text and in Fig. 1C.

Comment #25:
Fig 1D and lines 139-140: While the text indicates that there is a significant dose dependent effect of elacridar on the cells, there is no statistical comparison between elacridar doses in Fig 1D.

Response:

We have added the p-values (Tukey's multiple comparisons) on the effect of elacridar in Fig. 1D. All tested pairs show p<0.0001 (****), except for the 3 group pairs as marked in the figure.

Comment #26:
Fig 1D and associated methods: What is the transfection efficiency of the human MDR1 gene in HCT8 cells?

Response:

The transfection efficiency approaches 100%, as demonstrated by comparative immunostaining of HCT-8 cells transfected with MDR-carrying and blank vectors, utilizing identical antibody concentrations and exposure times (**S1 Fig**). This information is detailed in the Materials/Methods and Results sections.

Comment #27:

Fig 4F: In addition to the IFA, this fig shows a western blot (not mentioned in the figure legend) that I presume shows the anti-CpeIF4A reactivity to the sporozoites lysate. There are 1 major and 5 minor bands recognized in the blot calling into question the specificity of the antibody. How specific is this antibody for CpeIF4A?

Response:

New western blot analysis has been carried out, resolving the minor band issue. Now, a single band is recognized from the sporozoite extract. The minor bands in the previous image were likely due to protein degradation during sample preparation.

Comment #28:

Fig 6C. I cannot find a description or a reference for the anti-Cryptosporidium antibody used in this experiment.

Response:

The rabbit antibody was produced using a synthetic peptide as the immunogen. It is mentioned in the M&M section and explained in the Results section and figure legend.

Comment #29:

There are minor typo's throughout that need to be fixed. Ie: line 127: HapG2 should be HepG2; line 327: the IC50s have no units, Supplemental fig 3: titles should read meronts not merozonts

Response:

Thanks for careful reading of the manuscript. All typographical errors have been addressed. Additionally, the manuscript has undergone comprehensive revisions to enhance clarity and rectify textual issues.
